# Virtual Torque Sensor for Low-Cost RC Servo Motors Based on Dynamic System Identification Utilizing Parametric Constraints

**DOI:** 10.3390/s18113856

**Published:** 2018-11-09

**Authors:** Yoonkyu Hwang, Yuki Minami, Masato Ishikawa

**Affiliations:** Department of Mechanical Engineering, Osaka University, 2-1 Yamadaoka, Suita, Osaka 565-0871, Japan; minami@mech.eng.osaka-u.ac.jp (Y.M.); ishikawa@mech.eng.osaka-u.ac.jp (M.I.)

**Keywords:** virtual torque sensor, remote-controlled servo motor, internal dynamic model, system identification

## Abstract

We propose a novel virtual torque sensor for commercial low-cost radio-controlled (RC) servo motors. The virtual torque sensor has played an important role for conventional robots. It has been used for torque-required control applications such as human–robot interaction and under-actuated robots. However, most virtual torque sensors are based on the inversion of actuators or robot dynamics with the assumption that entire dynamics are known. This is not applicable to the RC servo motors that have unknown control structures. As RC servo motors enable researchers and hobbyists to create lightweight but high performance robots in an easy and cost-effective manner, the development of a virtual torque sensor for these motors is necessary. In this study, we propose a design method of a virtual torque sensor for RC servo motors. First, the virtual sensor is derived mathematically based on internal dynamic models with parametric constraints and compared to the conventional model. Second, a dedicated system identification method is developed for the proposed virtual sensor to implement the sensor in actual experiments. Finally, we compare experimental results with the measurements obtained by an actual sensor.

## 1. Introduction

Radio-controlled (RC) servo motors are compact actuators that are originally equipped with a sensor (potentiometer), a torque transmission system (gearbox), and a built-in controller (embedded board). These motors have been adopted by numerous researchers and hobbyists to realize lightweight but high performance robots using a simple interface in a cost-effective manner. For example, the motors were successfully utilized by aerial manipulators, hand exoskeletons, humanoid necks, worm robots, and cheetah robots in [1,2,3,4,5], respectively.

However, the limitation of RC servo motors is that they can be controlled only by the desired angle position [6,7]. Contrary to the conventional position-based control of robots, torque-based control provides a more convenient and reliable method for active applications such as human–robot interaction and under-actuated robotics. In recent years, as many robots are required to perform tasks under confined environments to interact safely with surroundings [8], the estimation of exerted torque is the most important part for RC servo motors to use them consistently as robot actuators in a variety of applications.

Two candidate approaches have been studied to solve the lack of torque information . One is to directly measure exerted torque by attaching physical sensors on robots (e.g., [9,10,11,12,13]). The other approach, which is referred to as the virtual sensor approach, is to indirectly estimate torque by exploiting mathematical system models. In [10], a robot is controlled by torque that is directly measured by an actual torque sensor. Even though the torque sensor is precise and can be installed on a motor shaft, the sensor is bulky or expensive. This nullifies the major advantages of RC servo motors. Torque can also be calculated by a current sensor with a current–torque proportional relationship, as in [11]. Unfortunately, it is difficult to install a current sensor on RC servo motors without the intrusive opening of packaging because current is managed by the inner microcontroller in the packaging. Opening the packaging is not preferred because it is tedious and it affects the pre-aligned gear system, which is related to hysteresis behavior. Although a few RC servo motors, called servo drives or smart servos, are known to provide torque information calculated by manufacturer, there are several issues left as follows. The first is on the reliability of the built-in torque sensor, whether it is accurate indeed for each product and various experimental situation. The problem may require experiments-based system identification process. In addition, it should be noted that there are many conventional robots using the RC servo motors that have no torque information. To the best knowledge of authors, there are various products in the market for the RC servo motors having no internal torque information. In this respect, the development of virtual torque sensor which is applicable to most kinds of RC servo motors provides greater flexibility not only for choosing actuators according to one’s budget and desired dimensions of the motors in purchase stage, but also for reusing conventional robots to the torque-required applications. On the contrary, a virtual sensor can estimate exerted torque without direct measurement if the governing system model is provided. The electromechanical dynamics of DC motors were studied for system modeling and control in [14]. In [15], a Kalman filter was designed for torque estimation based on a dynamic model of low-cost DC motors. The framework of a virtual torque sensor was implemented and validated for industrial robots [16,17], rehabilitation robots [18], and series elastic actuators in [19]. However, all these approaches assume that control structures are configurable or known by end users, which is not applicable to low-cost commercial RC servo motors with unknown control structures.

To the best of our knowledge, there have been few attempts to design torque observers considering system dynamics and unknown control structures. In [20], a continuous-time system identification procedure is presented for RC servo motors under blind control architecture based on the model complexity criterion. However, the study focuses on the validity for the determination of controller structure. In addition, there is insufficient explanation about how the identified models and torque are related in systematic manner, whether results are reasonable in the physical sense, and how it can be generally applied and expanded for more cases. In addition, all results for the proposed approach depend on the identified models that do not consider parametric constraints in dynamic models. This contradicts the assumption made by the study.

In this study, we propose a design method of a virtual torque sensor for commercial RC servo motors under an unknown control structure. First, the virtual torque sensor is derived based on internal dynamic models. Second, for the proposed virtual torque sensor framework, dedicated system identification is presented with the problem formulation of constrained optimization. Finally, experiments are conducted to validate the proposed virtual torque sensor. The torques estimated by our virtual torque sensor and an actual torque sensor are compared, and the result shows that the proposed sensor successfully estimates exerted torque.

The rest of this paper is organized as follows: In Section 2, the hardware configuration and internal dynamic model of RC servo motors are described. In Section 3, the problem formulation is presented with the system identification method. In Section 4, the experimental setup is illustrated and results are analyzed. The conclusions are presented in Section 5.

## 2. Virtual Torque Sensor for RC Servo Motors Using Dynamical System Model

In this section, we mathematically derive the virtual torque sensor for RC servo motors based on the internal dynamic model. First, the operation flow in RC servo motors is explained, followed by a brief description of the electromechanical dynamics for the internal dynamic model. Finally, we compare the proposed framework with the conventional framework and suggest a possible method to improve the robustness and precision of results.

**Figure 1:**
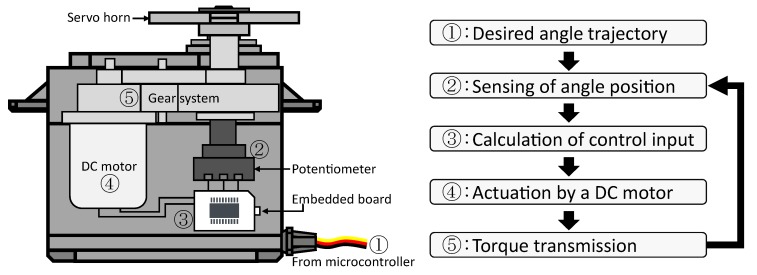
Schematic of an RC servo motor.

RC servo motors are operated in the following five steps: First, the desired angle trajectory is sent to a small built-in board from a microcontroller via a pulse width modulation duty signal. Second, the board receives the current angle position as proportional voltage through a potentiometer. Third, the board calculates the control input required to achieve the desired angle trajectory. Fourth, the DC motor is actuated by the torque exerted by the control voltage. Fifth, the generated torque is transmitted to the load shaft on which the potentiometer is placed. Finally, the above-mentioned steps are repeated with a fixed sample time. The steps are illustrated in Figure 1.

### 2.1. Electromechanical Model for DC Motors

**Figure 2:**
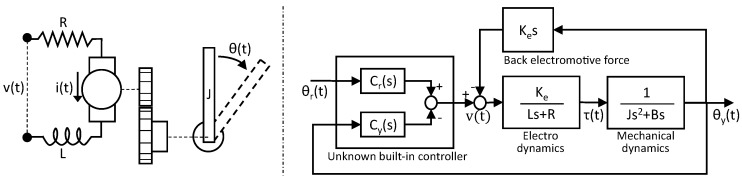
Block diagram for the system model of RC servo motor.

The electronic dynamics and mechanical dynamics are derived using the net current balance law and net torque balance law, respectively, as illustrated in Figure 2.

(1) $$\begin{array}{c} v\left(t\right)=Ri\left(t\right)+L\frac{di(t)}{dt}+K_{e}\dot{\theta }\left(t\right), \end{array}$$

(2) $$\tau \left(t\right)=J\ddot{\theta }\left(t\right)+B\dot{\theta }\left(t\right)=K_{e}i\left(t\right).$$

where *R*, *L*, and $K_{e}$ represent resistance, inductance and back-electromotive force, respectively, and *J*, *B*, and θ represent load inertia, viscous load friction, and angle, respectively. Then, for a single DC motor, the transfer functions from applied voltage to exerted torque and from exerted torque to the angle position of the motor are derived as follows:

(3) $$\frac{T(s)}{V(s)}=\frac{K_{e}\left(Js+B\right)}{\left(Ls+R\right)\left(Js+B\right)+K_{e}^{2}},$$

(4) $$\frac{\Theta (s)}{T(s)}=\frac{1}{Js^{2}+Bs}.$$

### 2.2. Torque Estimation under Unknown Control Structure

Exerted torque can be estimated by Equations (2)–(4) if the required inputs are provided. However, end users cannot measure the input voltage of the DC motor in Equation (3) because the voltage is adjusted by the unknown embedded controller in the enclosed case of the servo motor. In addition, internal current cannot be used to estimate exerted torque for the input of Equation (3) because the current sensor cannot be attached without the intrusive opening of servo motor packaging. The problem in using Equation (4) for the estimation of exerted torque is that the available data for end users are not from exerted torque to the output angle but from the reference angle to the output angle. This makes it difficult for end users to estimate the parameters in Equation (4) when two different dynamics exist. The unknown embedded controller and limited measurements are the main reasons the conventional virtual torque sensor cannot be implemented directly.

In this respect, first, we assume that the signal through the embedded controller is approximated by the linear combination of two source signals with arbitrary compensation terms.

(5) $$v\left(t\right)=C_{r}\left(s\right)\theta_{r}\left(t\right)-C_{y}\left(s\right)\theta \left(t\right).$$

Then, according to the block diagram in Figure 2 and Equation (5), exerted torque is

(6) $$T\left(s\right)=\frac{K_{e}}{Ls+R}\left((V\left(s\right)-K_{e}s\Theta \left(s\right)\right),$$

(7) $$T\left(s\right)=\frac{K_{e}}{Ls+R}\left(C_{r}\left(s\right)\Theta_{r}\left(s\right)-C_{y}\left(s\right)\Theta \left(s\right)-K_{e}s\Theta \left(s\right)\right).$$

Let $M\left(s\right)=\frac{\Theta (s)}{\Theta_{r}\left(s\right)}$ be the transfer function of the closed-loop system from reference angle position $\theta_{r}\left(t\right)$ to output angle position θ(t). Dividing Equation (7) by Θ(s) and using Equation (4),

(8) $$\begin{array}{cc} \frac{T(s)}{\Theta (s)} & =\frac{K_{e}}{Ls+R}\left(C_{r}\left(s\right)\frac{1}{M(s)}-C_{y}\left(s\right)-K_{e}s\right),\\ & =Js^{2}+Bs. \end{array}$$

At this step, more information is required to remove the explicit dependency on the arbitrary controller terms in Equation (8).

### 2.3. Virtual Torque Sensor with Parametric Constraints in Independent Experiments

To resolve the unknown controller terms in Equation (8), we propose an experimental method using selective parameter variation. A key part of the method is to design at least two independent experiments that show significantly different responses in the Laplace domain. In this method, multiple independent experiments are performed for different values of inertia $J_{i}$, where subscript *i* represents the *i*th experiment. In the same manner, $M_{i}\left(s\right)$ indicates the closed-loop system for the *i*th experiment. First, the virtual torque sensor is derived for two independent experiments (i=1,2). The generalization for more independent experiments is described at the end of this section. As the selective parameter variation for RC servo motors, chaining inertia *J* is a reasonable selection owing to the following three reasons: First, load inertia has a direct relationship with the exerted torque to be determined. Second, the effects of parameter variation in the mechanical system are more evident on output angle responses. Third, load inertia can be adjusted easily in most practical applications. Load inertia is changed with a fixed value, and other system models remain the same . Then, based on Equation (8), the system equation given below is satisfied for the two independent experiments.

(9) $$\begin{array}{c} \frac{K_{e}}{Ls+R}\left[\begin{array}{cc} \frac{1}{M_{1}\left(s\right)} & 1\\ \frac{1}{M_{2}\left(s\right)} & 1 \end{array}\right]\left[\begin{array}{c} C_{r}\left(s\right)\\ -C_{y}\left(s\right)-K_{e}s \end{array}\right]=\left[\begin{array}{c} J_{1}s^{2}+Bs\\ J_{2}s^{2}+Bs \end{array}\right]. \end{array}$$

Reformulating Equation (7) to the matrix form, the arbitrary controller terms in the torque equation are replaced with two closed-loop systems and inertia values.

(10) $$\begin{array}{cc} T(s) & =\frac{K_{e}}{Ls+R}\left[\begin{array}{cc} C_{r}\left(s\right) & -C_{y}\left(s\right)-K_{e}s \end{array}\right]\left[\begin{array}{c} \Theta_{r}\left(s\right)\\ \Theta (s) \end{array}\right],\\ & ={\left[{\left[\begin{array}{cc} \frac{1}{M_{1}\left(s\right)} & 1\\ \frac{1}{M_{2}\left(s\right)} & 1 \end{array}\right]}^{-1}\left[\begin{array}{c} J_{1}s^{2}+Bs\\ J_{2}s^{2}+Bs \end{array}\right]\right]}^{T}\left[\begin{array}{c} \Theta_{r}\left(s\right)\\ \Theta (s) \end{array}\right],\\ & ={\left[\begin{array}{c} -\frac{J_{2}-J_{1}}{\frac{1}{M_{1}}-\frac{1}{M_{2}}}s^{2}\\ J_{1}s^{2}+Bs+\frac{\frac{1}{M_{1}}}{\frac{1}{M_{1}}-\frac{1}{M_{2}}}\left(J_{2}-J_{1}\right)s^{2} \end{array}\right]}^{T}\left[\begin{array}{c} \Theta_{r}\left(s\right)\\ \Theta (s) \end{array}\right]. \end{array}$$

The inversion in Equation (10) exists only if $M_{1}\left(s\right)\neq M_{2}\left(s\right)$. This implies that the above terms can be used if the estimated closed-loop systems must not be the same. In addition, it is preferred to estimate two system models that are considerably different in the Laplace domain for the reliability of the inversion process. Using the reference angle position, output angle position, and Equation (10), the torque driven by the motor is calculated by

(11) $$\tau \left(t\right)=\Phi {\left(s\right)}^{-1}\theta \left(t\right)+\Delta \left(s\right)\left(\hat{\theta }\left(t\right)-\theta \left(t\right)\right),$$

where $\Phi \left(s\right)=\frac{1}{J_{1}s^{2}+Bs}$, $\Delta \left(s\right)=-\frac{J_{2}-J_{1}}{\frac{1}{M_{2}\left(s\right)}-\frac{1}{M_{1}\left(s\right)}}\frac{s^{2}}{M_{1}\left(s\right)}$, and $\hat{\theta }\left(t\right)=M_{1}\left(s\right)\theta_{r}\left(t\right)$. Torque τ(t) is composed of three terms, i.e., prediction $\Phi {\left(s\right)}^{-1}\theta \left(t\right)$, the error between the prediction and actual value $\left(\hat{\theta }\left(t\right)-\theta \left(t\right)\right)$, and error compensation Δ(s). Δ(s) compensates prediction error by extrapolating between the change in inertia and the induced closed-loop responses. This is a major difference from conventional virtual sensors. It should also be noted that the prediction term is an improper transfer function and acts as a second-order differentiator. In practical applications, the term is highly sensitive to measurement noise. To reduce the influence of noise, a low-pass filter can be used on the output response at the expense of estimation delay. Finally, exerted torque can be estimated as $\hat{\tau }\left(t\right)$ using the low-pass filter, F(s).

(12) $$\hat{\tau }\left(t\right)=\left(-\frac{J_{2}-J_{1}}{\frac{1}{M_{1}}-\frac{1}{M_{2}}}s^{2}\right)\theta_{r}\left(t\right)+\left(J_{1}s^{2}+Bs+\frac{\frac{1}{M_{1}}}{\frac{1}{M_{1}}-\frac{1}{M_{2}}}\left(J_{2}-J_{1}\right)s^{2}\right)F\left(s\right)\theta \left(t\right).$$

The proposed method can be expanded for more than two experiments if more robust estimation is desired.

(13) $$\left[\begin{array}{cc} \frac{1}{M_{1}\left(s\right)} & 1\\ \frac{1}{M_{2}\left(s\right)} & 1\\ \vdots & \vdots \\ \frac{1}{M_{n}\left(s\right)} & 1 \end{array}\right]\left[\begin{array}{c} C_{r}\left(s\right)\\ -C_{y}\left(s\right)-K_{e}\left(s\right) \end{array}\right]=\left[\begin{array}{c} J_{1}s^{2}+Bs\\ J_{2}s^{2}+Bs\\ \vdots \\ J_{n}s^{2}+Bs \end{array}\right].$$

Then, the virtual torque sensor can be obtained by applying pseudo-inversion or regression methods, followed by the procedure described in this section.

## 3. System Identification for the Proposed Virtual Torque Sensor

This section presents the system identification method for the unknown parameters or system models in Equation (12). After the constraints in internal dynamics are addressed, the identification process is formulated as an optimization problem with an objective function and constrained conditions in the frequency domain.

### 3.1. System Identification in the Lumped Parameters

To implement the proposed virtual torque sensor, the terms of $M_{1}\left(s\right),M_{2}\left(s\right),J_{2}-J_{1},J_{1},andB$ must be identified or provided for at least two significantly different experiments. Based on the block diagram in Figure 2, the closed-loop system model is

(14) $$M_{i}\left(s\right)=\frac{K_{e}C_{r}\left(s\right)}{\left(J_{i}s+B\right)\left(Ls+R\right)s+K_{e}^{2}s+K_{e}C_{y}\left(s\right)},i\in \left\{1,2\right\}.$$

Signals through controllers are approximated by the outputs of candidate transfer functions and validated using experimental data. This is presented in the later part of this paper. One of the candidate structures for the output of controllers is a polynomial expansion in s, that is, $C_{r}\left(s\right)=C_{y}\left(s\right)=\frac{b(s)}{a(s)}$, where $a\left(s\right)=s^{n}+a_{n-1}s^{n-1}+\cdots +a_{0}$ and $b\left(s\right)=b_{m}s^{m}+b_{m-1}s^{m-1}+\cdots +b_{0}$. Then, the closed-loop system, $M_{i}\left(s\right)$, is

(15) $$\begin{array}{cc} M_{i}\left(s\right) & =\frac{K_{e}b\left(s\right)}{s\left(J_{i}s+B\right)\left(Ls+R\right)a\left(s\right)+K_{e}^{2}a\left(s\right)s+K_{e}b\left(s\right)},\\ & =\frac{K_{e}\left(b_{m}s^{m}+\cdots +b_{0}\right)}{JLs^{n+3}+\left(BL+JR\right)s^{n+2}a_{n-1}+\cdots +K_{e}b_{m}s^{m}+\cdots +K_{e}b_{0}}. \end{array}$$

In the identification process, the parameters in $M_{i}\left(s\right)$ are estimated with normalized coefficients set as ${\hat{p}}_{i}=\left[p_{0}^{i},p_{1}^{i},\cdots ,p_{n_{+}m+3}^{i}\right]$ for identifiability [21].

(16) $$\hat{M_{i}}\left(s\right)=\frac{p_{m}^{i}s^{m}+p_{m-1}^{i}s^{m-1}+\cdots +p_{0}^{i}}{s^{n+3}+p_{n+m+3}^{i}s^{n+2}+p_{n+m+2}^{i}s^{n+1}\cdots +p_{m+1}^{i}}.$$

Comparing Equation (15) with Equation (16), the relationships given below are satisfied for the cases of m<n+2

(17) $$\begin{array}{cc} \frac{J_{2}}{J_{1}} & =\frac{p_{m}^{1}}{p_{m}^{2}},\\ B & =\frac{J_{1}J_{2}}{J_{2}-J_{1}}\left(p_{n+m+3}^{1}-p_{n+m+3}^{2}\right). \end{array}$$

Thus, all unknowns in the torque estimator can be identified from closed-loop experiments with a known value of ($J_{2}-J_{1}$). Furthermore, in Equation (17), there are terms that are independent of different values of inertia $J_{i}$. For example, the cases shown in Table 1 for the case of *n* = 1 and *m* = 1.

Then, constrained conditions are generated between the coefficients of the estimated models.

(18) $$\frac{p_{i}^{1}}{p_{1}^{1}}-\frac{p_{i}^{2}}{p_{1}^{2}}=0,\;\forall i\in \left[0,2,3\right].$$

### 3.2. Problem Formulation as Constrained Optimization in Frequency Domain

**Figure 3:**
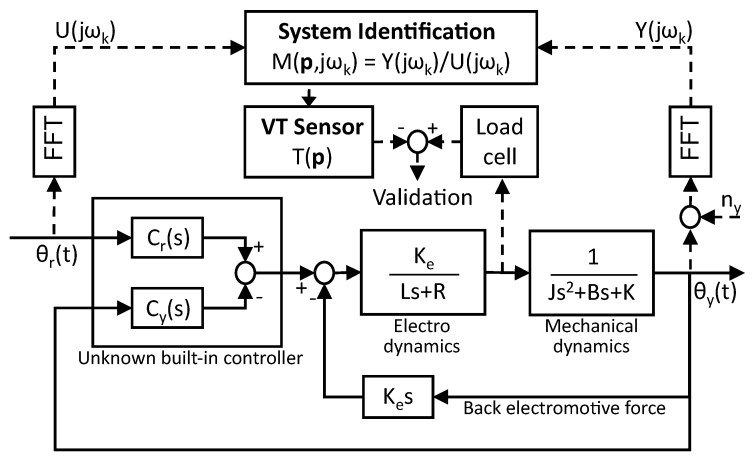
Frequency-domain identification scheme for the proposed virtual torque sensor.

Frequency-domain system identification is a mature identification method of designing experiments and analyzing results in a systematic manner. The identification flow for the proposed virtual torque sensor is illustrated in Figure 3. First, after the system is excited by the designed reference angle position signals, output angle positions are measured and saved with the input signal in the time domain. Second, input–output data are transformed to the set of frequency data through the fast Fourier transform, which reduces the influence of noise in the measured output. Third, to estimate the unknown parameters in the system model, optimization is performed for the set of input–output frequency data. The main aim of the identification is to minimize the prediction error between frequency-domain data, $M_{i}^{*}\left(j\omega_{k}\right)$, and the output of the estimated model, $M_{i}\left(j\omega_{k},{\hat{p}}_{i}\right)$. Therefore, the unknown parameters, $\hat{p}=\left[{\hat{p}}_{1},{\hat{p}}_{2}\right]$, in Equation (16) can be estimated by the objective function, $f(\hat{p})$, as

(19) $$f\left(\hat{p}\right)=\min_{\hat{p}}\sum_{i=1}^{2}{\left(\sum_{k=1}^{n_{f}}{\left(\frac{|M_{i}^{*}\left(j\omega_{k}\right)\left|-\right|M_{i}\left(j\omega_{k},{\hat{p}}_{i}\right)|}{W_{i}\left(k\right)}\right)}^{2}\right)}^{\frac{1}{2}},$$

$$\begin{array}{cc} \mathrm{subject}\;\mathrm{to}\; & g_{1}\left(\hat{p}\right):=\max\left\{Re\left[\lambda_{l}\left(M_{i}\left(s,\hat{p}\right)\right)\right]\right\}<0,\forall i,l,\\ & g_{2}\left(\hat{p}\right):=p_{0}^{1}p_{1}^{2}-p_{0}^{2}p_{1}^{1}=0,\\ & g_{3}\left(\hat{p}\right):=p_{2}^{1}p_{1}^{2}-p_{2}^{2}p_{1}^{1}=0,\\ & g_{4}\left(\hat{p}\right):=p_{3}^{1}p_{1}^{2}-p_{3}^{2}p_{1}^{1}=0. \end{array}$$

Weighting value $W_{i}\left(k\right)$ represents the standard deviation of the repeated experiments at the *k*th frequency out of $n_{f}$ frequencies. Constraint $g_{1}\left(\hat{p}\right)$ is created for the stability of the closed loop when $\lambda_{i}$ is the *i*th eigenvalue of the estimated closed-loop system, $M_{i}\left(s,\hat{p}\right)$, and constraints $g_{2}\left(\hat{p}\right),g_{3}\left(\hat{p}\right)$, and $g_{4}\left(\hat{p}\right)$ are introduced by the internal dynamic model. In this problem, the equality constraints are handled by expressing an unknown variable using known variables in the equation. Then, the constrained optimization problem, $f(\hat{p})$, can be transformed to the unconstrained problem, $h(\hat{p})$, by reformulating the objective function and constraints as shown below. A detailed explanation can be found in [22].

(20) $$\min_{\hat{p}}h\left(\hat{p}\right)=\left\{\begin{array}{cc} g_{1}\left(\hat{p}\right) & \mathrm{if}\;\;g_{1}\left(\hat{p}\right)\geq 0,\\ f(\hat{p}) & \mathrm{otherwise} \end{array}\right..$$

A meta-heuristic algorithm, cyclic network topology-based PSO [23], is used to solve the optimization problem given by Equation (20). Let $x_{\alpha }^{\beta }$ be the αth particle of a swarm at the βth iteration, where each $x_{\alpha }^{\beta }$ represents one hypothesis for the optimal result of $\hat{p}$. Parameters $L_{p}$, $n_{p}$, and $n_{s}$ denote the length of the parameter vector to be found, the number of particles at an iteration, and the number of neighbors of the αth particle, respectively.

Step 0 (Initialization) 

All particles are randomly sampled from the uniform distribution. The best previously obtained position (pbest) and the best position in the social neighborhood (sbest) are set.

$$\begin{array}{c} x_{\alpha }^{0}\in {ℜ}^{L_{p}},\alpha =1,2,\ldots ,n_{p},\\ x_{\alpha ,pbest}^{0}\overset{}{\leftarrow }x_{\alpha }^{0},\\ x_{\alpha ,sbest}^{0}\overset{}{\leftarrow }\arg\min_{x\in \{x_{\gamma }^{0}|\gamma =\alpha -\frac{n_{s}}{2},\ldots ,\alpha +\frac{n_{s}}{2}\}}h(x). \end{array}$$

Step 1 (Optimal Solution) 

The optimization is terminated if the optimal value, $x^{*}$, satisfies the desired level.

(21) $$x^{*}:=\arg\min_{x\in \{x_{\beta }^{\alpha }|\alpha =1,\ldots ,n_{p},\alpha =0,\ldots ,k\}}h(x).$$

Otherwise, Step 2 is performed.

Step 2 (Evolutionary Update) 

All particles are moved by the following evolutionary update law:

(22) $$x_{\alpha }^{k+1}\overset{}{\leftarrow }c_{0}x_{\alpha }^{k}+c_{1}r_{1,\alpha }^{k}\left(x_{pbest,\alpha }^{k}-x_{\alpha }^{k}\right)+c_{2}r_{2,\alpha }^{k}\left(x_{sbest,\alpha }^{k}-x_{\alpha }^{k}\right).$$

User-designed parameters $c_{0},c_{1},$ and $c_{2}$ represent the inertia factor, cognitive scaling factor, and social scaling factor, respectively. The values of pbest and sbest are updated as given below. Then, Step 1 is performed.

(23) $$\begin{array}{c} x_{pbest,\alpha }^{k}\overset{}{\leftarrow }\arg\min_{x\in \{x_{\alpha }^{\beta }|\beta =1,2,\ldots ,k\}}h(x),\\ x_{sbest,\alpha }^{k}\overset{}{\leftarrow }\arg\min_{x\in \{x_{\alpha }^{n_{iter}}|\alpha =\beta -\frac{n_{s}}{2},\ldots ,\beta +\frac{n_{s}}{2}\}}h(x). \end{array}$$

The experimental setup and results are described in the next section.

## 4. Experiments and Analysis

This section describes the identification experiments conducted to specify the proposed virtual torque sensor. The unknowns in the proposed sensor are resolved based on the result of the closed-loop system identification from the reference angle position to the output angle position. The performance of the virtual torque sensor is validated using actual torque data.

### 4.1. Experimental Setup

**Figure 4:**
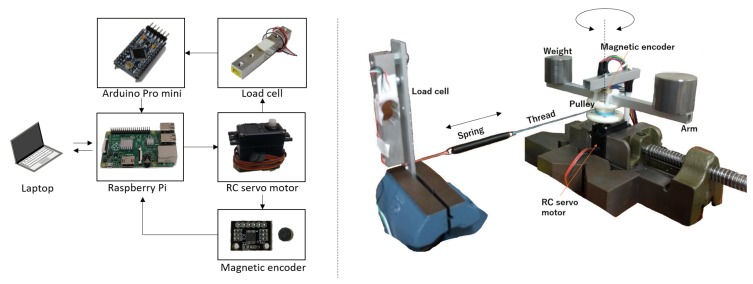
Schematic of experimental setup.

The experimental setup is illustrated in Figure 4. A load cell is used for validation to compare exerted torque with actual values. A pulley with a spring and a thread connected by the load cell is installed. Owing to this, the mechanical dynamics shown in Figure 2 are slightly changed from $\frac{1}{Js^{2}+Bs}$ to $\frac{1}{Js^{2}+Bs+K}$, where K is the spring stiffness constant. Calibrated weights are placed on a lightweight arm fixed on the pulley. A microrotary encoder is introduced to measure the current angle position. The specifications of the equipment used in the experiments are provided in Table 2.

### 4.2. Identification of the Proposed Torque Estimator

The performance of result models depends on the design of the input excitation signal. It is known that the input signal should be carefully created for the target situation. Otherwise, the identified model experiences undesired influences such as leakage effects or nonlinear effects [21]. In the experiments, we select random multisine sets as excitation signals because the sets have beneficial properties such as bounded amplitude, no leakage effects, and well-defined randomness to nonlinear effects. Multisine inputs are realized based on Equation (24) for each independent experiment, which is repeated seven times.

(24) $$u\left(t\right)=\sum_{k=1}^{n_{f}}Usin\left(\omega_{k}t+\varphi_{k}\right).$$

where $n_{f}$ is the number of desired frequencies, $w_{k}$ is the desired frequency, *U* is a constant, and $\varphi_{k}$ is a random phase that is uniformly distributed in the interval $\left[0,2\pi \right]$. Parameters U=0.0425, $n_{f}=25$, and $w_{k}$ are selected from 0.1 Hz to approximately 10 Hz, satisfying an equal interval on the log scale. The input can be used to estimate up to 50 order of a model in an unambiguous way because the persistent excitation order of the multisine inputs is 50.

**Figure 5:**
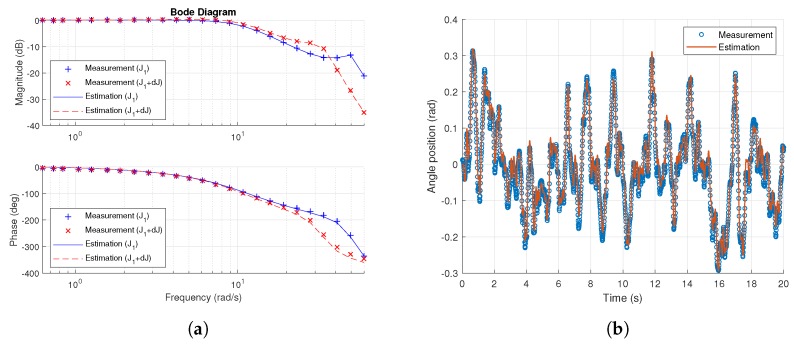
Results of system model identification from reference angle to output angle. (**a**) Comparison between measurements and the estimated model in frequency domain; (**b**) Comparison between measurements and the estimated model in time domain for initial inertia of $J_{1}$.

Input–output datasets are measured in the time domain and transformed to frequency data for calibrated weights of 200 g and 450 g. The parameters identified in the result models ${\hat{M}}_{i}\left(s,\hat{x}\right)$ by averaging the seven repeated experiments are shown in Table 3, and the estimated responses are compared with the experimental responses in Figure 5.

$${\hat{M}}_{i}\left(s,\hat{x}\right)=\frac{p_{1}^{i}s+p_{0}^{i}}{s^{4}+p_{5}^{i}s^{3}+p_{4}^{i}s^{2}+p_{3}^{i}s+p_{2}^{i}},$$

$$\begin{array}{cc} \mathrm{subject}\;\mathrm{to}\; & g_{1}\left(\hat{x}\right)=\max\left\{Re\left[\lambda_{l}\left(M_{i}\left(s,\hat{x}\right)\right)\right]\right\}=-6.4488<0,\forall i,l,\\ & g_{2}\left(\hat{x}\right)=p_{0}^{1}p_{1}^{2}-p_{0}^{2}p_{1}^{1}=0,\;g_{3}\left(\hat{x}\right)=p_{2}^{1}p_{1}^{2}-p_{2}^{2}p_{1}^{1}=0,\;g_{4}\left(\hat{x}\right)=p_{3}^{1}p_{1}^{2}-p_{3}^{2}p_{1}^{1}=0. \end{array}$$

Considering that the inertia difference, $J_{2}-J_{1}$, is $1.75\times 10^{-4}$ based on the calibrated weights and arm length, $J_{1}$ and *B* are calculated using Equation (16). In addition, parameter *K* can be found by solving the system of equations introduced by Table 4.

(25) $$\left\{\begin{array}{c} x_{3}+x_{4}+\frac{B}{J_{i}}-p_{5}^{i}=0\\ \frac{B}{J}x_{3}+\frac{B}{J}x_{4}+x_{3}x_{4}+\frac{1}{J_{i}}x_{1}+\frac{1}{J_{i}}x_{2}-p_{4}^{i}=0\\ \frac{1}{J_{i}}x_{1}x_{3}+\frac{1}{J_{i}}x_{1}x_{4}+\frac{1}{J_{i}}x_{2}x_{4}+\frac{B}{J_{i}}x_{3}x_{4}+p_{1}^{i}-p_{3}^{i}=0\\ \frac{1}{J_{i}}x_{1}x_{3}x_{4}+p_{0}^{i}-p_{2}^{i}=0 \end{array}\right.$$

where $\left[x_{1},x_{2},x_{3},x_{4}\right]=\left[K,\frac{K_{e}^{2}}{L},\frac{R}{L},a_{0}\right]$. It is numerically solved by employing the trust-region dogleg algorithm. The solution is $\left[x_{1},x_{2},x_{3},x_{4}\right]=\left[0.2244,0.1291,25.6764,-0.1179\right]$. Then, $\left[J_{1},B,K\right]=\left[1.2347\times 10^{-4},4.7722\times 10^{-4},0.2244\right]$. All unknowns in Equation (12) are resolved by the system identification. Finally, exerted torque is estimated by the proposed virtual torque sensor (i.e., Equation (12)) with $F\left(s\right)=\frac{1}{0.5s^{2}+s+1}$.

(26) $$\hat{\tau }\left(t\right)=\frac{-0.4932s+40.07}{s^{2}+25.56s+339.1}\theta_{r}\left(t\right)+\frac{-0.174s^{2}+1.343s+73.386}{s^{4}+27.558s^{3}+392.178s^{2}+729.239s+678.122}\theta \left(t\right).$$

**Figure 6:**
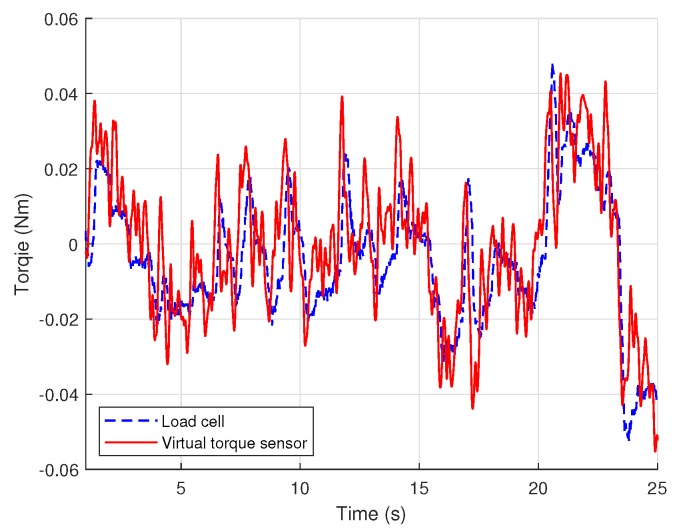
Validation of the performance of virtual torque sensor using the measurement of load cell.

The estimated torque is compared with the measurement obtained from load cell data in Figure 6. The torque value by the load cell was not used for any identification process. The result proves that the proposed virtual torque sensor successfully estimates the true torque exerted by the RC servo motor.

## 5. Conclusions

In this study, we investigated a virtual torque sensor for commercial RC servo motors under an unknown control structure. The proposed virtual sensor was designed, implemented, and validated using the following procedure: First, the mathematical model of the virtual sensor was derived without explicit dependency on the arbitrary controller. Based on the internal dynamic models of RC servo motors, the controllers were substituted by closed-loop systems under a specially designed condition. Second, the problem of parameter identification in the closed-loop systems was formulated as a constrained optimization problem in the frequency domain and solved by utilizing the meta-heuristic optimization algorithm. Finally, experimental results proved that our virtual sensor successfully approximates the torque values measured by an actual physical sensor. The proposed virtual torque sensor can be easily used for low-cost applications or small-sized prototype robots equipped with RC servo motors without an actual torque sensor.

## Figures and Tables

**Table 1:** Coefficients of $\hat{M_{i}}\left(s\right)$ for n=1 and m=1.

Coefficient	Value	Coefficient	Value
$p_{0}^{i}$	$\frac{K_{e}b_{0}}{J_{i}L}$	$p_{3}^{i}$	$\frac{K_{e}b_{1}+K_{e}^{2}a_{0}+BRa_{0}}{J_{i}L}$
$p_{1}^{i}$	$\frac{K_{e}b_{1}}{J_{i}L}$	$p_{4}^{i}$	$\frac{K_{e}^{2}}{J_{i}L}+\frac{BR}{J_{i}L}+\frac{Ba_{0}}{J_{i}}+\frac{Ra_{0}}{L}$
$p_{2}^{i}$	$\frac{K_{e}b_{0}}{J_{i}L}$	$p_{5}^{i}$	$\frac{B}{J_{i}}+\frac{R}{L}+a_{0}$

**Table 2:** Specifications of experimental equipment.

Equipment/Item	Model	Description
RC servo motor	GWS S03T 2BBMG	Max torque: 7.93 kf/cm
Microrotary encoder	AS5048A	Resolution: 14 bits
Microcontroller	Raspberry Pi 3 Model B+	Real-time control with 1.4 GHz 64-bit
Analog to digital converter	Arduino Pro Mini 328 3.3 v 8 MHz	Resolution: 10 bits
Load cell		Force range: 0–1 kg

**Table 3:** Identified parameters of $\hat{M_{i}}\left(s\right)$ for n=1 and m=1.

Coefficient	Value	Coefficient	Value	Coefficient	Value	Coefficient	Value
$p_{0}^{1}$	−3.9949 $\times \;10^{3}$	$p_{3}^{1}$	4.2319 $\times \;10^{4}$	$p_{0}^{2}$	−1.6526 $\times \;10^{3}$	$p_{3}^{2}$	1.7506 $\times \;10^{4}$
$p_{1}^{1}$	3.2451 $\times \;10^{5}$	$p_{4}^{1}$	2.9589 $\times \;10^{3}$	$p_{1}^{2}$	1.3424 $\times \;10^{5}$	$p_{4}^{2}$	1.4228 $\times \;10^{3}$
$p_{2}^{1}$	3.1901 $\times \;10^{5}$	$p_{5}^{1}$	29.4236	$p_{2}^{2}$	1.3196 $\times \;10^{5}$	$p_{5}^{2}$	27.1573

**Table 4:** Coefficients of $\hat{M_{i}}\left(s\right)$ for n=1 and m=1 for the identified model.

Coefficient	Value	Coefficient	Value
$p_{0}^{i}$	$\frac{K_{e}b_{0}}{J_{i}L}$	$p_{3}^{i}$	$\frac{K_{e}b_{1}+K_{e}^{2}a_{0}+BRa_{0}+KR+KLa_{0}}{J_{i}L}$
$p_{1}^{i}$	$\frac{K_{e}b_{1}}{J_{i}L}$	$p_{4}^{i}$	$\frac{K_{e}^{2}}{J_{i}L}+\frac{BR}{J_{i}L}+\frac{K}{J_{i}}+\frac{Ba_{0}}{J_{i}}+\frac{Ra_{0}}{L}$
$p_{2}^{i}$	$\frac{K_{e}b_{0}+KRa_{0}}{J_{i}L}$	$p_{5}^{i}$	$\frac{B}{J_{i}}+\frac{R}{L}+a_{0}$
